# Addressing COVID-19 vaccine hesitancy in rural community pharmacies: a protocol for a stepped wedge randomized clinical trial

**DOI:** 10.1186/s13012-023-01327-7

**Published:** 2023-12-18

**Authors:** Geoffrey Curran, Cynthia Mosley, Abigail Gamble, Jacob Painter, Songthip Ounpraseuth, Noel T. Brewer, Ben Teeter, Megan Smith, Jacquie Halladay, Tamera Hughes, J. Greene Shepherd, Tessa Hastings, Kit Simpson, Delesha Carpenter

**Affiliations:** 1 Center for Implementation Research, Department of Pharmacy Practice, University of Arkansas for Medical Sciences, Little Rock, AR USA; 2 Eshelman School of Pharmacy, University of North Carolina, Chapel Hill, NC USA; 3 Pharmaceutical Evaluation and Policy, Department of Pharmacy Practice, University of Arkansas for Medical Sciences, Little Rock, AR USA; 4 Department of Biostatistics, University of Arkansas for Medical Sciences, Little Rock, AR USA; 5 Department of Health Behavior, Gillings School of Global Public Health, University of North Carolina, Chapel Hill, NC USA; 6 Lineberger Comprehensive Cancer Center, University of North Carolina, Chapel Hill, NC USA; 7 Department of Family Medicine, University of North Carolina, Chapel Hill, Chapel Hill, NC USA; 8 The Cecil G. Sheps Center for Health Services Research, University of North Carolina, Chapel Hill, NC USA; 9 Department of Clinical Pharmacy and Outcomes Sciences, College of Pharmacy, University of South Carolina, Columbia, SC USA; 10 Department of Health Care Leadership and Management, College of Health Professions, Medical University of South Carolina, Charleston, SC USA; 11 Department of Public Health Science, College of Medicine, Medical University of South Carolina, Charleston, SC USA

**Keywords:** Implementation strategies, Vaccine hesitancy, COVID-19, Community pharmacies, Rural, Implementation science, Communication

## Abstract

**Background:**

Uptake of COVID-19 vaccines remains problematically low in the USA, especially in rural areas. COVID-19 vaccine hesitancy is associated with lower uptake, which translates to higher susceptibility to SARS-CoV-2 variants in communities where vaccination coverage is low. Because community pharmacists are among the most accessible and trusted health professionals in rural areas, this randomized clinical trial will examine implementation strategies to support rural pharmacists in delivering an adapted evidence-based intervention to reduce COVID-19 vaccine hesitancy.

**Methods:**

We will use an incomplete stepped wedge trial design in which we will randomize 30 rural pharmacies (unit of analysis) to determine the effectiveness and incremental cost-effectiveness of a standard implementation approach (consisting of online training that describes the vaccine hesitancy intervention, live webinar, and resource website) compared to adding on a virtual facilitation approach (provided by a trained facilitator in support of the delivery of the vaccine hesitancy counseling intervention by pharmacists). The intervention (ASORT) has been adapted from an evidence-based vaccine communication intervention for HPV vaccines through a partnership with rural pharmacies in a practice-based research network in seven southern US states. ASORT teaches pharmacists how to identify persons eligible for COVID-19 vaccination (including a booster), solicit and address vaccine concerns in a non-confrontational way, recommend the vaccine, and repeat the steps later if needed. The primary trial outcome is fidelity to the ASORT intervention, which will be determined through ratings of recordings of pharmacists delivering the intervention. The secondary outcome is the effectiveness of the intervention, determined by rates of patients who agree to be vaccinated after receiving the intervention. Other secondary outcomes include feasibility, acceptability, adoption, reach, and cost. Cost-effectiveness and budget impact analyses will be conducted to maximize the potential for future dissemination and sustainability. Mixed methods will provide triangulation, expansion, and explanation of quantitative findings.

**Discussion:**

This trial contributes to a growing evidence base on vaccine hesitancy interventions and virtual-only facilitation of evidenced-based practices in community health settings. The trial will provide the first estimate of the relative value of different implementation strategies in pharmacy settings.

**Trial registration:**

NCT05926544 (clinicaltrials.gov); 07/03/2023.

Contributions to the literature • This trial will capture intervention and implementation outcomes associated with the delivery of a COVID-19 vaccine hesitancy counseling intervention in rural community pharmacies.

• Cost-effectiveness and budget impact analyses will compare the relative value of two implementation approaches (standard alone vs. standard *plus* virtual facilitation) and provide feasibility estimates for rural pharmacies to implement the intervention.

• A mixed methods evaluation will examine contextual factors associated with implementation and the sustainability potential of using virtual facilitation to support the delivery, with fidelity, of a counseling intervention in rural pharmacies.

## Background

Major health crises like the COVID-19 pandemic have disproportionally affected rural communities. Rural populations in the USA have a high prevalence of factors, including older age, obesity, and other health conditions that put them at greater risk for COVID-19 complications, including hospitalization and death [1]. Indeed, by the close of a summer surge of the Delta variant approximately 18 months into the pandemic (October 2021), the COVID-19 death rate in rural areas was more than double that of urban areas [2]. Rural communities are frequently health professional shortage areas that lack healthcare infrastructure, including hospitals [3–5], which complicates their ability to treat severe COVID-19 illness. Thus, *prevention* is an important part of a comprehensive strategy for protecting rural populations from the negative impact of COVID-19.

When compared to urban populations, individuals living in rural areas are more vaccine-hesitant [6–8]. Vaccine hesitancy is defined as a “motivational state of being conflicted about or opposed to getting vaccinated” [9]. People with higher vaccine hesitancy are much less likely to be vaccinated [10]. After a year of COVID-19 vaccine availability in the USA, rural populations had one of the lowest percentages of vaccine uptake (62%) and the second highest percentage of people who said they would “definitely not” get vaccinated (20%) [6]. Regarding drivers of vaccine hesitancy, rural residents are more likely to feel that the vaccine is unsafe and believe vaccine myths [11], and are less likely to use or have access to trustworthy information sources [5, 11]. Hence, it is imperative to implement COVID-19 vaccine hesitancy interventions in rural communities.

As one of the most accessible health professionals in rural areas [12–14], community pharmacists are a notable exception to the lack of rural healthcare infrastructure. The USA has over 68,000 community pharmacies, many of which have convenient hours and offer free walk-in services, making them more accessible for individuals who do not have health insurance [15]. Pharmacists are also one of the most trusted sources of medication information [16] and can serve as strong allies in addressing vaccine hesitancy. Rural patients see their community pharmacist ~ 14 times per year; nearly three times more than they see a primary care provider [17]. Thus, pharmacists can address vaccine concerns frequently and make repeated vaccination offers. For pharmacists to maximize the delivery of vaccinations [18], they need guidance on how to address vaccine hesitancy [19] and updated information to address patients’ evolving vaccine concerns as well as implementation support, including training and ongoing guidance, to deliver evidence-based vaccine hesitancy counseling interventions [20, 21].

Implementation science, defined as the “study of methods to promote the systematic uptake of… evidence-based practices into routine practice,” can guide efforts to optimally support pharmacists to engage in vaccine hesitancy counseling [22]. Testing of *implementation strategies* [23], which are discrete interventions or activities that support the successful uptake of evidence-based practices/interventions, is key to understanding how to best support pharmacists as they implement sensitive vaccine hesitancy conversations [24]. A common combination of implementation strategies to help health professionals implement new practices typically involves training, implementation guides with steps, and other implementation support tools (e.g., reminders, sample language to use with patients). This “standard approach” is usually not sufficient to promote the adoption of a new and complex practice with fidelity [25–27]. In recognition of this limitation, a growing body of research shows that implementation facilitation can increase practice/intervention fidelity across a wide range of clinical contexts [28–35] by having trained facilitators build trusting relationships with health professionals to monitor their implementation progress, provide feedback, and reinforce change. Of note, a recent study found that a facilitation strategy increased primary care clinics’ adoption of an HPV vaccine announcement intervention threefold (63% vs 16%) when compared to standard HPV communication training alone [36].

A recent systematic review of implementation strategies in community pharmacies found that the least studied or used implementation strategy was “providing interactive assistance,” or as described above, facilitation*.* Despite the growing positive results of the effects of facilitation on implementation success, little systematic research on facilitation has been conducted with community pharmacies. Even less is known about delivering facilitation virtually, whereby facilitators connect with health professionals exclusively via telephone and video. The need to study virtual facilitation is highly relevant given that travel to remote rural locations is resource-intensive.

## Specific aims

The overall objective is to test the effects of a *standard implementation* approach and the addition of *virtual facilitation* on rural pharmacists’ ability to implement COVID-19 vaccine hesitancy counseling, using an incomplete stepped wedge design [37]. Data will be collected on the primary trial outcome of counseling fidelity (competence) and the secondary outcome of intervention effectiveness (vaccination rates). Pharmacies will implement the ASORT intervention, which was adapted from an evidence-based vaccine hesitancy intervention [38] with extensive qualitative input from rural pharmacists [39]. The intervention will be updated frequently to address new vaccine concerns as they arise.

### Specific aim 1

Compared to the standard implementation approach, test whether adding virtual facilitation increases (a) the fidelity with which pharmacists implement the vaccine hesitancy counseling intervention and (b) the number of vaccine hesitant patients who receive the vaccine. Our *primary outcome* is pharmacist fidelity to the vaccine hesitancy counseling intervention, and our *secondary outcome* is intervention effectiveness. We hypothesize that virtual facilitation improves fidelity to the intervention and intervention effectiveness when compared to standard implementation.

### Specific aim 2

Conduct a cost assessment and explore the potential sustainability of the implementation approaches. We will conduct time-driven, activity-based cost-effectiveness analysis and budget impact analyses to estimate the relative value and feasibility of standard implementation and virtual facilitation approaches. Additionally, a payer advisory committee (PAC) will review these data and advise on how to make virtual facilitation sustainable through reimbursement models.

## Methods

We will use a randomized clinical trial with an adapted stepped-wedge design and 30 rural pharmacies to determine the effectiveness and incremental cost-effectiveness of a standard implementation approach compared to the addition of virtual facilitation by a trained facilitator in support of the delivery of a COVID-19 vaccine hesitancy counseling intervention. Mixed methods will provide triangulation, expansion, and explanation of quantitative findings.

Following well-respected implementation science recommendations [40, 41], we used multiple implementation frameworks to guide our proposed research. Specifically, the *Proctor Taxonomy of Implementation Outcomes* [42] guided our selection of trial outcomes. It describes conceptually distinct implementation outcomes including feasibility, acceptability, appropriateness, adoption, fidelity, and cost, as well as subsequent “service outcomes” including the effectiveness of clinical/preventive interventions. The qualitative data collection and analysis plans were guided by the Proctor framework (for triangulation and explanation) as well as the *Consolidated Framework for Implementation Research* (CFIR), which provide an understanding of continued implementation challenges and potential barriers/facilitators for the sustainability of the intervention and the two implementation approaches. CFIR, which borrows constructs from Rogers’ Diffusion of Innovations and other sources [43, 44], organizes multiple constructs influencing the implementation and sustainment of practices into five domains: intervention characteristics, outer setting, inner setting, characteristics of individuals, and process. The cost evaluation plans were informed by Cidav et al. [45] who laid out a time-driven, activity-based costing approach for implementation research. Preliminary research to adapt the vaccine hesitancy intervention and tailor implementation strategies was informed by Evidence-Based Quality Improvement (EBQI), a participatory approach [46] to prepare for implementation. EBQI brings together clinical and implementation experts with local providers, decision-makers, and key stakeholders to adopt evidence-based practices for context and to select and operationalize implementation strategies [29, 47–51].

### Trial setting

All data will be collected from rural pharmacies that participate in the Rural Reseach Alliance of Community Pharmacies (RURAL-CP), which was established by this trial’s multiple principal investigators Carpenter and Curran in 2020 [52]. RURAL-CP is the first practice-based research network for rural community pharmacies and is registered with the Agency for Healthcare Research and Quality. The mission of RURAL-CP is to reduce rural health disparities by supporting high-quality implementation research with community pharmacies. At the time of the proposal’s submission, RURAL-CP consisted of 111 rural community pharmacies from five southeastern states (Alabama, Arkansas, Mississippi, North Carolina, and South Carolina) and included a mix of independent, regional chain, and small grocery store pharmacies, ranging from isolated non-metro rural areas to larger, micropolitan rural areas [53]. RURAL-CP has since expanded to seven states and 127 pharmacies (recruitment of new pharmacies ongoing).

### Sample

We will recruit 30 RURAL-CP pharmacies. In order to target rural communities with high levels of vaccine hesitancy, 15 pharmacies will be recruited from counties that have African American populations ≥ 25.1%, which is the mean percentage of African Americans in RURAL-CP counties in 2020 according to the U.S. Census, and 15 pharmacies will be recruited from counties that had at least 64.9% vote for a Republican president in 2020, which is the mean percentage of individuals who voted for a Republican in RURAL-CP counties. We have selected this recruitment strategy because both African American and Republican/conservative populations have consistently had very high levels of vaccine hesitancy [7, 54, 55]. Pharmacies will be invited to participate initially via email and before randomization. RURAL-CP-affiliated state leads will do follow-up calls if emails generate no response. Trial MPIs (Curran and Carpenter) will obtain informed consent over the phone with pharmacy leads. If a pharmacy declines participation or “drops out” after initially enrolling, we will replace it with a randomly selected RURAL-CP pharmacy from the same or similarly qualifying county.

### Vaccine hesitancy counseling intervention

The ASORT intervention will ask pharmacists to identify and engage in COVID-19 vaccine hesitancy counseling with one to two vaccine-hesitant individuals weekly. As noted earlier, we have refined an evidence-based vaccine hesitancy counseling intervention with extensive feedback from rural pharmacists. These refinements resulted in a 5-step counseling process (ASORT; see Table 1) as well as an online resource that provides example verbiage for over 25 vaccine concerns that have been expressed in rural communities. The vaccine hesitancy verbiage is updated periodically with input from our rural pharmacist (four RURAL-CP pharmacists) and rural patient (four rural patients; two African American, two with Republican party affiliation) advisory boards.

**Table 1:** Selected content from the 5-step vaccine hesitancy counseling process (ASORT)

**Step**	**Recommendations and example verbiage**
**A**sk if they would like to receive a COVID vaccination	• People are more open to talking about the COVID-19 vaccine if you ask while you’re doing other activities, like giving a flu shot or engaging in medication therapy management. *“While I’m giving your flu shot, I just thought I’d ask if you’ve gotten your COVID -19 vaccine yet.”* • Offer praise to people who are up-to-date on their vaccination
**S**olicit their main vaccine concern	• People often have multiple concerns about the vaccine, but one concern will likely loom larger than the others, so this is the concern you’ll want to focus on first. *“Can you tell me more about that?”*
**O**ffer to address their concerns	• People have different levels of readiness to discuss the vaccine, so it’s important to ask for permission to share more information about their concerns. • Start by validating their concerns so they know that you’re not judging them. *“I know several other people who have had that same concern and I’ve shared some information with them that they’ve found useful. I’d be happy to share that same information with you if you want.”* • Some people won’t be ready for more information and that’s okay. Just let them know that you understand. *“Ok. No problem. Know that I’m here if you do ever want to talk.”* • Address their concerns • For individuals who aren’t ready, skip to the last step.
**R**ecommend the vaccine	• Share your personal experience with the vaccine and that you trust it before you recommend it. This can help build their trust in the vaccine. • After sharing your personal experience, then recommend the vaccine. *“I wouldn’t recommend the vaccine if I didn’t think it was safe. I received it and I trust it. That’s why I recommend that you get the vaccine - because I care about you and want you to keep you safe.”* • You can also tie your recommendation to any factors that may put them or their family members at higher risk for severe COVID-19 complications. • If they are still unsure or refuse, then move to the next step.
**T**ry again later if they refuse or are unsure	• As we’ve seen throughout the pandemic, many people who say they will never get the vaccine have since been vaccinated. So don’t be discouraged if they refuse. React in a positive way and let them know you’ll check in with them again. *“Thanks for considering it. I’ll check in with you again if I hear any new information about your concern.”* • Since people can and do change their minds, it’s important to try again during one of their next visits to the pharmacy. • For regular customers, keep a list of people to follow up with or make a note in the pharmacy record to follow up.

### Implementation strategies

The standard approach will train and prepare pharmacists to implement ASORT and provide discrete implementation support tools to support intervention fidelity. Specifically, a *trial website* will include numerous tools, including example vaccine hesitancy verbiage, sample workflows, marketing materials, and patient pamphlets. The standard approach also includes an *online training module* developed by the trial team that incorporates similar instructional design principles that have been used previously to develop pharmacist communication-focused modules [56, 57]. Finally, just prior to the start time of each block of pharmacies, participants will attend a *live webinar with continuing education (CE) credit* that includes interactive training on the intervention, updated vaccination recommendations, vaccine storage and delivery, and documentation.

**Fig. 1:**
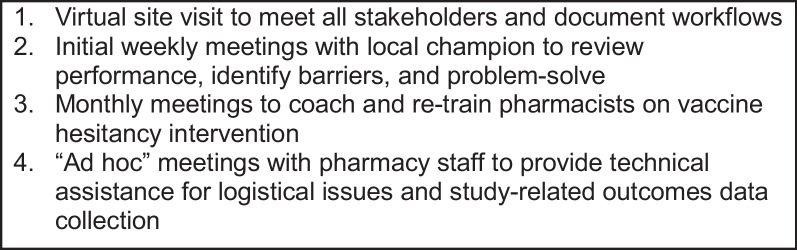
Summary of virtual facilitator schedule of activities

The *virtual facilitation* approach will provide expert guidance from trained facilitators regarding intervention content and implementation processes. The facilitators will perform the following evidence-supported functions [58–62]: engaging stakeholders; building relationships; identifying and training a local facilitator/champion; monitoring progress; providing feedback on progress; identifying implementation barriers; problem-solving; re-training and coaching; and reinforcing change (see Fig. 1). Facilitators will attend a 16-h virtually-delivered training in implementation facilitation provided by the *Implementation Facilitation Learning Hub*, a training center supported by the U.S. Department of Veterans Affairs (VA) Quality Enhancement Research Initiative (QUERI). The training teaches principles and techniques contained in Dollar et al.’s manual *Using Implementation Facilitation to Improve Healthcare (Version 3* [61];), which was developed by the VA Behavioral Health QUERI, a research center devoted to supporting the implementation of behavioral health interventions with the VA. The training is highly interactive [63], involving significant practice and role play. Training topics include knowledge, skills, core competencies of facilitators; facilitation roles and activities (e.g., assessing the site, engaging stakeholders, problem identification and resolution); phases of implementation; delivering facilitation virtually; and evaluating facilitation. After the training, Dr. Curran (trial co-principal investigator) will provide ongoing coaching and supervision to the trial facilitators.

### Aim 1: data collection and measures

Both quantitative and qualitative data will be collected. We will use Qualtrics (Provo, UT) to collect aggregated, de-identified data on effectiveness (the secondary outcome) and adoption. Research staff will complete assessments of fidelity (the primary outcome) and costs (time and activity logs subsequently converted to costs). At the end of the standard and virtual facilitation periods, pharmacy staff from each pharmacy will complete surveys to assess the other implementation outcomes listed in Table 2. We also will select one high and one low-performing pharmacy from each block of our stepped-wedge design and conduct interviews with at least four pharmacy staff (pharmacists and pharmacy technicians) per pharmacy, immediately after the virtual facilitation period has ended, to provide context for the quantitative outcomes.

**Table 2:** Outcome measure descriptions and data collection procedures

**Measure**	**Description**	**Quantitative**	**Qualitative**^**a**^
Primary outcome			
Fidelity	Degree to which the vaccine hesitancy counseling intervention was delivered as intended	Fidelity Checklist that assesses adherence and competence to vaccine hesitancy counseling intervention. Each pharmacist assessed twice per month.	Interviews with pharmacy personnel
Secondary outcome			
Effectiveness	The proportion of vaccine-hesitant individuals who vaccinate after receiving hesitancy counseling	Monthly report completed by pharmacy designee using web-based reporting system	Interviews with pharmacy personnel
Other measures			
Acceptability	Stakeholder perceptions regarding satisfaction with and appeal of the vaccine hesitancy intervention and implementation approaches	4-item measure completed by pharmacy personnel at end of standard and virtual facilitation approach [95]	Semi-structured interviews with pharmacy personnel
Uptake	How often vaccine hesitancy counseling was offered to hesitant individuals	Monthly report completed by pharmacy designee using web-based reporting system	
Appropriateness	Stakeholder perceptions regarding fit and suitability of the vaccine hesitancy intervention and implementation approaches	4-item measure completed by pharmacy personnel at end of standard and virtual facilitation approach [95]	
Feasibility	Stakeholder perceptions regarding ease and “do-ability” of the vaccine hesitancy intervention and implementation approaches	4-item measure completed by pharmacy personnel at the end of the standard and virtual facilitation approach [95]	
Organizational Structure and Context	Key structural aspects of pharmacy (e.g., size/staffing) and organizational capacity for change	Structure survey completed by one pharmacist before randomization; context survey completed by ≥ 5 pharmacy personnel	N/A
Sustainment	Continued measurement of fidelity, effectiveness, and uptake during the “follow-up” periods	See above entries for fidelity, effectiveness, and uptake	
Cost	Costs associated with deployment of each implementation approach.	Log of time and activities completed monthly by facilitator [93]	

^a^Qualitative data will be collected two months after the end of the virtual facilitation period with 2 high performing and 2 low performing pharmacies per block

### Aim 1: quantitative outcome measures

*Fidelity* (the primary outcome) to the vaccine hesitancy counseling intervention will be assessed at the pharmacy level. Our fidelity measure is based on a theoretical framework of fidelity measurement [64] as well as a validated fidelity checklist [65]. The fidelity measure focuses on the competence of the pharmacist (7 items) in their delivery of the vaccine hesitancy counseling intervention.

The competence items focus on the skillfulness of intervention delivery: expressed empathy; used a non-confrontational manner; spoke confidently without using jargon; emphasized patient autonomy; reflected back patient’s statements accurately; used a respectful demeanor, and used evidence-based responses when responding to patient vaccine concerns. Each competence item will be assessed on a scale from 0 to 2, with 0 = skill not demonstrated, 1 = skill needs development, and 2 = skill demonstrated with competence. Competence scale scores will range from 0 to 14, with higher scores reflecting greater competency in the delivery of ASORT. Fidelity will be measured for each pharmacist approximately twice per month under the standard implementation approach and approximately twice per month under the virtual facilitation approach. In pharmacies with more than one pharmacist, fidelity ratings will be averaged to achieve a pharmacy-level measure.

Trained staff will rate fidelity after reaching 80% inter-rater reliability during training [66]. Staff who are blinded to the pharmacist’s group assignment will use a fidelity observation guide to rate pharmacists’ vaccine hesitancy counseling during recorded counseling sessions. Pharmacists will be instructed to seek permission from patients to audio record all interventions until two are recorded and submitted each month. Patients will provide verbal consent before the session is recorded. Previous research has demonstrated that recording health professionals’ communication does not alter their communication behavior [67, 68]. A HIPAA-compliant phone application will be used to allow the pharmacists to record the interventions on their own phones, securely send the recordings to a trial team member, and then delete the recordings once they have been rated. During virtual facilitation, ratings will be shared with the facilitators who provide feedback and coaching to pharmacists towards improving fidelity.

Effectiveness (the secondary outcome) will be assessed on a monthly basis. Pharmacists will be instructed to deliver vaccine hesitancy counseling to one to two vaccine-hesitant individuals each week (towards a target of at least 5 per month). Using Qualtrics, pharmacists will document the following on a daily basis:

(A) How many vaccine-hesitant individuals they provided vaccine hesitancy counseling to;
(B) Of those individuals, how many received a COVID-19 vaccine;
(C) The self-reported age, race, and gender of the individual who was counseled.

*Effectiveness* will be calculated as the proportion of counseled individuals who received a vaccine, or B/A. Individuals who are counseled and schedule a vaccine at a later date will not be counted in the numerator (B) until they have actually received a vaccine. Effectiveness scores will range from 0 to 1, with higher scores reflecting a greater percentage of vaccine-hesitant individuals who received a vaccine. During virtual facilitation, trial facilitators will be provided with bi-weekly effectiveness data so they can share the results with the pharmacies to assess performance and identify ways to improve implementation processes and intervention effectiveness.

### Other measures

*Acceptability, appropriateness, and feasibility* of the vaccine hesitancy intervention and implementation approaches (standard, facilitation) will be measured using validated surveys [69]. Each measure includes 4 items (e.g., “intervention *X* seems doable” [feasibility]) measured on a 5-point response scale that ranges from “completely disagree” (coded as 1) to “completely agree” (coded as 5). For each pharmacy, we will average scores across the five pharmacy staff members who complete the surveys.

- *Uptake* will be calculated as the number of times vaccine hesitancy counseling was offered divided by the number of individuals who expressed vaccine hesitancy when offered the vaccine.
- *Sustainment* will be assessed by the continued measurement of *fidelity*, *effectiveness*, and *uptake* during the “follow-up” periods.
- *Cost* measures are described below.
- *Organizational structure and context measures* [70]: one pharmacist per pharmacy will complete the Organizational Structure survey [71, 72], which measures: location, type (e.g., independent, chain), setting (e.g., retail, specialty), size (weekly prescriptions, staffing), technological capacity (dispensing system), and services provided. The Organizational Context measure (completed by 5 pharmacy staff members) assesses CFIR inner setting constructs [70] that reflect organizational culture/capacity for change [73], learning climate, leadership, and resources [74, 75]. All items are measured on a 5-point agree-disagree response scale, with higher scores reflecting a stronger implementation context. For each pharmacy, we will average scores across the five pharmacy staff members who complete the surveys.

### Aim 1: randomization

**Fig. 2:**
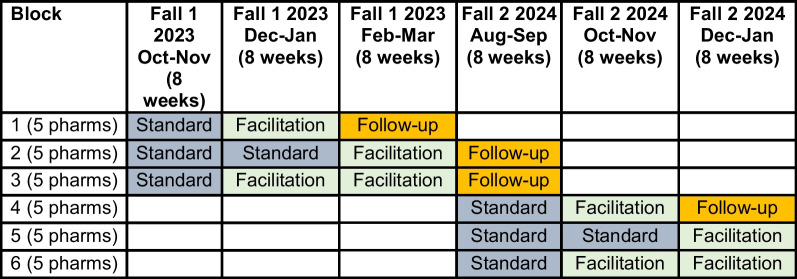
Incomplete stepped wedge randomized trial

In this stepped-wedge trial, pharmacies will be randomized in blocks at the time they begin the initial intervention condition (standard implementation). We will create 6 blocks of pharmacies, with 5 pharmacies randomized to each block by the trial statistician (Ounpraseuth). Figure 2 depicts the “stairstep” [76] pharmacy assignment and randomization plan. We will employ two-stage randomization—first to a block and then to a start time. To ensure balance, the block randomization will be stratified by two measures of pharmacy size—the number of patients and number of pharmacists (which are reported on the organizational structure survey). Each block will participate in either one or two 8-week standard implementation periods followed by one or two 8-week virtual facilitation periods. For blocks 1–4, we will continue to collect data during one 8-week follow-up period to evaluate the potential impact of the virtual facilitation approach on sustained intervention fidelity and effectiveness once virtual facilitation has been stopped.

The design described here is a modification of our original design. We had to revise the design to follow the expected future distribution of COVID-19 vaccines in the USA, which will follow the Northern Hemisphere’s “flu season”—approximately August-February each year. We expect that the first COVID-19 vaccination season in the fall of 2023 will be delayed due to vaccine availability and will begin in October and run through March 2024. In 2024, we expect that availability issues will not occur and the vaccine will be available during the standard flu season from August-February. The original design built on “year-round” availability of vaccine instead of seasonal or intermittent availability, with 3 blocks of 10 pharmacies receiving 4 months of standard implementation, 4 months of virtual facilitation, and at least one 4-month period of follow-up.

This unavoidable change in design comes with potential positive and negative impacts. A potential negative effect is the need to reduce the timeframe of each implementation observation period from 4 to 2 months. This potential negative impact is counteracted by the fact that the ASORT intervention is focused and brief, and that impacts of the standard and virtual facilitation approaches can be observed within two months’ time. Further, the adjustment to the flu season calendar allows us to explore the relative impacts of receiving the standard implementation and virtual facilitation approaches for 2 *or* 4 months, which has helpful implications, especially for the cost-effectiveness analysis. The design change negatively impacts plans to assess implementation sustainment in follow-up periods. However, we are still able to assess for evidence of *immediate* sustainment across two blocks of pharmacies (1 and 4), while assessing for evidence of *lagged* sustainment across two blocks of pharmacies (2 and 3). Immediate sustainment will be measured in the 8-week period immediately following receipt of virtual facilitation, possible only for pharmacies in blocks 1 and 4. Lagged sustainment will be measured within the initial 8 weeks of vaccine availability *in the next “season,”* possible only for blocks 2 and 3 within the timeline and budget constraints. The proposed assessment of sustainment was already a secondary outcome, but the design change allows only a preliminary look at sustainability potential of the two implementation approaches.

### Aim 1: data analysis

We will use the principles of intention-to-treat, using multiple imputations if needed for missingness, for all statistical analyses related to primary and secondary endpoints. Descriptive statistics will be computed for all quantitative implementation outcomes in Table 2. The primary outcome of fidelity is captured at the pharmacy level. For our primary analysis of the fidelity outcome, we will use linear mixed-effects models (LMM) to compare the level of fidelity between the two implementation approaches (standard approach vs. virtual facilitation). We will report point estimates for the group mean difference along with a 95% confidence interval. The model-building approach will follow four analyses steps: (1) an unadjusted before/after of the effect of the virtual facilitation approach (ignoring period/time effect); (2) the time period (i.e., steps/blocks) to examine if any potential intervention effect relates only to the intervention or also to an independent effect of calendar time; (3) an adjustment for potential pharmacy-level confounders, such as size and learning climate; and (4) the interaction between period and intervention effect.

For the secondary outcome of effectiveness, we will use generalized linear mixed models (GLMM) to investigate whether pharmacies are more effective at addressing vaccine hesitancy during virtual facilitation when compared to standard implementation. The effectiveness outcome will be binary (vaccine-hesitant patient accepts vaccine after counseling = 1; vaccine-hesitant patient does not accept vaccine after counseling = 0). We will report the odds ratio estimate of accepting the vaccine after counseling for the virtual facilitation approach (facilitation versus standard) along with a 95% confidence interval. The model-building approach for our secondary effectiveness outcome will follow four analysis steps: (1) an unadjusted before/after of the effect of the virtual facilitation approach (ignoring period/time effect); (2) the inclusion of time period (i.e., steps) to examine if any potential intervention effect relates only to the intervention or also to an independent effect of calendar time; (3) an adjustment for patient’s age, race, and gender and potential pharmacy-level confounders, such as pharmacy size; (4) the possible interaction between time period and intervention. The impact of virtual facilitation on effectiveness could potentially change over time if vaccine acceptance rates increase with time and as pharmacists gain experience with counseling vaccine-hesitant patients. We aim to explore this question through the inclusion of an interaction between period/time and intervention effect in Model 4. Additionally, the intraclass correlation coefficients (ICC) will be estimated and reported, so this information will be available for power analyses for future investigations using similar designs and outcomes. In order to reduce potential bias, during the virtual facilitation period, pharmacists will be instructed to approach patients that they did not approach during the standard facilitation period, which is feasible given the large number of unvaccinated individuals in the communities in which RURAL-CP pharmacies are located.

### Aim 1: power analyses

For the primary outcome of fidelity, captured at the pharmacy level, we wish to compare the fidelity scores under standard implementation versus fidelity scores under virtual facilitation. We expect each pharmacy to have approximately 4 fidelity assessments per time period (i.e., a period of 8 weeks). A sample of 30 pharmacies in an incomplete stepped-wedge cluster-randomized design with six periods (five steps), and an average of 10 fidelity assessments per pharmacy yields a total sample size of 320 assessments, which achieves over 90% power to detect a difference between means of 0.53 with a standard deviation of 1 (i.e., moderate effect size). The test statistic is based on a two-sided Wald Z-test with ICC = 0.6 and alpha = 0.05. Given that we will have repeated fidelity measures from the same pharmacists over time, we have specified a conservative ICC (which in a stepped wedge design does not impact power calculations significantly).

For the secondary outcome of effectiveness, we expect each pharmacy to identify approximately 10 vaccine-hesitant patients during each time period (i.e., a period of 8 weeks). A sample of 30 pharmacies in an incomplete stepped-wedge cluster-randomized design with six periods (five steps), and an average of 27 patients per pharmacy yields a total sample size of 800 patients, which achieves over 90% power to detect a difference between effectiveness proportions of 10%. The proportion of vaccine-hesitant patients accepting the vaccine during the virtual facilitation approach is assumed to be 15% compared to 5% under the standard implementation period. These estimates are based on a review of reported changes in vaccine acceptance for evidence-based vaccine hesitancy interventions [77]. The test statistic is based on a two-sided Wald *Z*-test with ICC = 0.05 and alpha = 0.05. We note that the actual sample size should exceed 800 patients since we expect pharmacies will continue to identify vaccine-hesitant individuals during the follow-up periods.

### Aim 1: qualitative data collection and analysis

We will select one high and one low-performing pharmacy in each block and conduct interviews after their virtual facilitation period has ended. Semi-structured interviews will be conducted [78] with at least 4 pharmacy staff members at each pharmacy, including pharmacy owners/managers, pharmacists, and technicians, to triangulate with and expand upon the quantitative implementation outcomes. Interview guides will be built around “grand tour” questions for each implementation outcome (Table 2), e.g., “How would you describe the implementation of vaccine hesitancy counseling?,” “What has gone well or not well?,” and “How feasible was it to receive facilitation as delivered?” Additionally, we will include questions to explore: (1) any observed associations between pharmacy size, patient demographics (race, age, gender), and organizational structure and context measures with fidelity and effectiveness; (2) sustainability potential; and (3) costs at the pharmacy level. Themes on sustainability and costs will be summarized and reported to the Payer Advisory Committee (PAC) during the sustainability exploration in Aim 2.

We will use rigorous procedures [79] for the analysis of qualitative interview data. Interviews will be transcribed by a professional transcriptionist, who will remove identifying information, and be imported into MAXQDA, a qualitative data analysis program. The trial team will review several transcripts and meet to discuss the overarching themes related to the CFIR framework (e.g., “not enough time” coded as an “inner context barrier”). These themes will then be incorporated into a codebook with definitions and example quotes to enable structured coding [80, 81]. Using rigorous analysis techniques [82], two researchers will use the codebook to independently code each interview and meet to resolve discrepancies. Inter-coder reliability will be calculated. Additionally, we will add specific attributes (e.g., number of scripts filled per day, pharmacy level of rurality) to each transcript, allowing us to examine whether fidelity and effectiveness vary by attribute. Because these interview guides and the quantitative data are both mapped to CFIR constructs, we will be able to conduct concurrent triangulation (comparing results from both data sources on the same questions) as well as elaboration analyses (using the qualitative data to provide depth of understanding to the quantitative findings) [83, 84].

### Aim 2: rationale, design, and sample

**Fig. 3:**
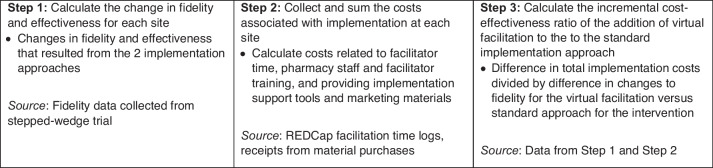
Analyses to examine cost-effectiveness of virtual facilitation vs. standard implementation approach

Cost-effectiveness analysis and budget impact analysis provide estimates of value and affordability, respectively. When used to examine the relative value and cost of implementation strategy bundles, these approaches inform decision-makers about the optimal approach to implementation and also whether that approach is financially feasible in a given setting. The calculation of the incremental cost-effectiveness ratio (ICER) comparing the relative value of implementation approaches for the vaccine hesitancy counseling intervention will be accomplished in three steps (Fig. 3).

### Aim 2: data analysis

For step 1, see the fidelity and effectiveness analysis described in the “Aim 1 data analysis” section. For step 2, implementation strategy costs for each site will be calculated using the data sources listed in Fig. 3. We have tested and found REDCap facilitator activity logs feasible in a prior study [61]. In step 3, the numerator will be the incremental difference in total implementation costs incurred at sites while receiving virtual facilitation compared to the standard implementation approach. The denominator will include the difference in the changes in fidelity and effectiveness while receiving virtual facilitation compared to the period receiving the standard implementation approach. Typical standard error estimation methods are not appropriate for cost-effectiveness ratios because the possibility of having a zero or near zero denominator is not negligible and cost and effectiveness estimates are rarely independent. Therefore, we will use a nonparametric bootstrap with a replacement method with 1000 replications to generate an empirical joint distribution of incremental implementation costs and fidelity and effectiveness change scores and acceptability curves representing the probability of falling below a range of cost-effectiveness thresholds identified by the PAC for an 8-point increase in fidelity and 10% increase in effectiveness. We will also conduct a budget impact analysis to provide an estimate of the cost for rural community pharmacies to implement the intervention. The budget impact analysis estimates the cost to implement and provide the intervention during the (a) standard implementation period and (b) virtual facilitation period for each site. Intervention costs will be estimated by multiplying the average time to complete the intervention by the number of times hesitancy counseling was provided. This time will be converted to costs using location-based salary and fringe estimates for the pharmacist. These estimates will be combined with implementation costs to estimate the budget impact of the intervention from the perspective of a pharmacy under each implementation condition

### Aim 2: sustainability exploration

In order to scale up new, effective interventions in the healthcare system, applied research efforts must engage key stakeholders, including payers, providers, and patients. The PAC will review data and prepare a business case and sustainability plan for implementing the ASORT intervention using the standard implementation and virtual facilitation approaches. Current ‘pain points’ and gaps that lead to reimbursement models that support the scalability and sustainability of the intervention and implementation approaches will be identified. At present, while pharmacists can receive a low administration fee for delivering vaccines, there is not yet a model of reimbursement for counseling. One such reimbursement pathway to explore is the North Carolina Department of Health and Human Services Collaborative Care (CoCM) codes [85], which are behavioral health codes for providers to share with other care providers. The PAC will comprise representatives from several health insurance payers, including Blue Cross and Blue Shield, and a provider organization to facilitate ‘team-based’ reimbursement strategies. The PAC will meet to reflect on the cost analysis results and develop feasible plans for reimbursement and sustainability of the implementation approaches.

## Discussion

To our knowledge, this randomized trial will be the first to evaluate a rural pharmacist-focused COVID-19 vaccine hesitancy counseling intervention. With extensive qualitative feedback from rural pharmacists, we refined an evidence-based vaccine hesitancy intervention to apply to rural pharmacy settings [39]. Preliminary data show that rural pharmacists rank patient concerns about COVID-19 vaccines as their top barrier to delivering the vaccine, so identifying the best methods to support pharmacist-delivered vaccine hesitancy counseling is innovative and timely. Through its implementation science lens, this trial will test the effects of novel and competing implementation approaches that could increase the cost-effective implementation of the COVID-19 vaccine hesitancy counseling intervention in rural pharmacies. How to best structure and provide facilitation in community pharmacies is currently unknown, although preliminary and pilot data [86] support the feasibility of virtual facilitation. If successful, the virtual facilitation approach could have far-reaching impacts since it can be adapted to support other evidence-based practices, such as point-of-care testing and various disease state management interventions, in community pharmacies. The mixed methods evaluation should identify how the ASORT intervention and implementation approaches can be improved to maximize their potential for future implementation and sustainment.

Examining the cost-effectiveness of implementation strategies is a critical next step to advance implementation science [87]. Specifically, researchers are tasked with identifying the cost of their implementation strategy relative to the benefit it provides to inform decisions on scaling the strategy [88, 89], which allows stakeholders to make informed decisions about resource allocation [90, 91]. In the context of implementation studies, cost-effectiveness analysis can be used to estimate differences in costs and implementation-specific outcomes such as fidelity between implementation strategies. In this trial, the cost-effectiveness and budget impact analyses should provide timely information needed to support the business case for widespread adoption of evidence-based practices in community pharmacies supported by standard implementation and virtual facilitation approaches.

The trial has potential challenges and limitations. As noted above, the largest challenge thus far has been the uncertainty around the scheduling and availability of COVID-19 vaccines. Indeed, even as of this protocol submission, the schedule has not been finalized and the trial design and timeline may have to be adjusted again. It is possible that it will be challenging to recruit and retain pharmacies. Given previous success in recruiting RURAL-CP pharmacies for research efforts, we expect to be able to recruit and retain 30 pharmacies successfully. Based on pharmacist recommendations, we have built in an incentive of up to $2500 for pharmacies to participate, which will cover their time to complete the standard approach training, participate in facilitation activities, document outcomes, and participate in qualitative data collection efforts. To mitigate potential social desirability bias, we will emphasize that we are evaluating the intervention, not the pharmacist. The nature of vaccine hesitancy is a “moving target,” which could complicate the trial. We will update our website and sample verbiage on a regular basis to stay current with reports from the field and published results on hesitancy concerns and points of discussion shown to overcome them. Most community pharmacies in the RURAL-CP network have been delivering the COVID-19 vaccine for over 2 years [21], so the logistical ordering and administration processes have already been established, though pharmacists can receive support for logistical issues during virtual facilitation if needed.

## Data Availability

The datasets used or analyzed during the trial will be available from the corresponding author upon reasonable request.

## Abbreviations

CFIR: Consolidated Framework for Implementation Research
EBQI: Evidence-Based Quality Improvement
RURAL-CP: Rural Research Alliance of Community Pharmacies
VA: U.S. Department of Veterans Affairs
QUERI: Quality Enhancement Research Initiative
LMM: Linear mixed-effects model
GLMM: Generalized linear mixed model
ICC: Intraclass correlation coefficients
ICER: Incremental cost-effectiveness ratio
PAC: Payer Advisory Committee
CoCM: Collaborative Care Management
